# Complete genome sequence of a member of a potentially novel genus within the family Opitutaceae isolated from a liquid medium used for co-cultivating duckweeds and river water-derived microbes

**DOI:** 10.1128/mra.00394-25

**Published:** 2025-10-16

**Authors:** Yosuke Morishita, Tomoki Iwashita, Manabu Kanno, Hideyuki Tamaki, Yoichi Kamagata, Tadashi Toyama, Kazuhiro Mori, Masaaki Morikawa, Yasuhiro Tanaka

**Affiliations:** 1 Graduate School of Engineering, University of Yamanashi 38146 Kofu, Yamanashi, Japan; 2 Bioproduction Research Institute, AIST 13508 , Tsukuba, Ibaraki, Japan; 3 Division of Biosphere Science, Graduate School of Environmental Science, Hokkaido University 12810, Sapporo, Hokkaido, Japan; 4 Graduate School of Life and Environmental Sciences, University of Yamanashi 38146 Kofu, Yamanashi, Japan; California State University San Marcos, San Marcos, California, USA

**Keywords:** Opitutaceae, bacterial genome

## Abstract

We report the complete genome sequence of strain MRC-7, which represents a potentially novel genus within the family Opitutaceae. The genome was 4,342,534 bp in size and contained 3,428 protein-coding sequences, several of which were predicted to be involved in pectin degradation.

## ANNOUNCEMENT

Aquatic plants are known to harbor taxonomically diverse microbes, including rarely cultivated groups (1–3). With this background, we developed the microbial isolation technique, “duckweed-microbe co-cultivation method,” which was effective for isolating rarely cultivated bacterial groups (4–6). Using this method, we successfully isolated strain MRC-7, a member of the fastidious bacterial family Opitutaceae, as follows (6): First, to prepare aseptic duckweed, turions were washed in 0.5% sodium hypochlorite for 5  min, followed by two rinses with sterile water. The treated turions were transplanted into modified Hoagland medium (7) for germination, and the germinated duckweed was subsequently used as aseptic duckweed. River water collected on August 30, 2021 (35.675270°N, 138.577518°E), was used as the microbial source and inoculated into the aseptic duckweed. Co-cultivation was carried out in modified Hoagland medium for 10 days (two 5- day batches). The resulting co-cultivated liquid medium was inoculated onto 1/100-strength tryptic soy agar plates and incubated at 25°C for 2 weeks, from which strain MRC-7 was isolated.

**Fig 1:**
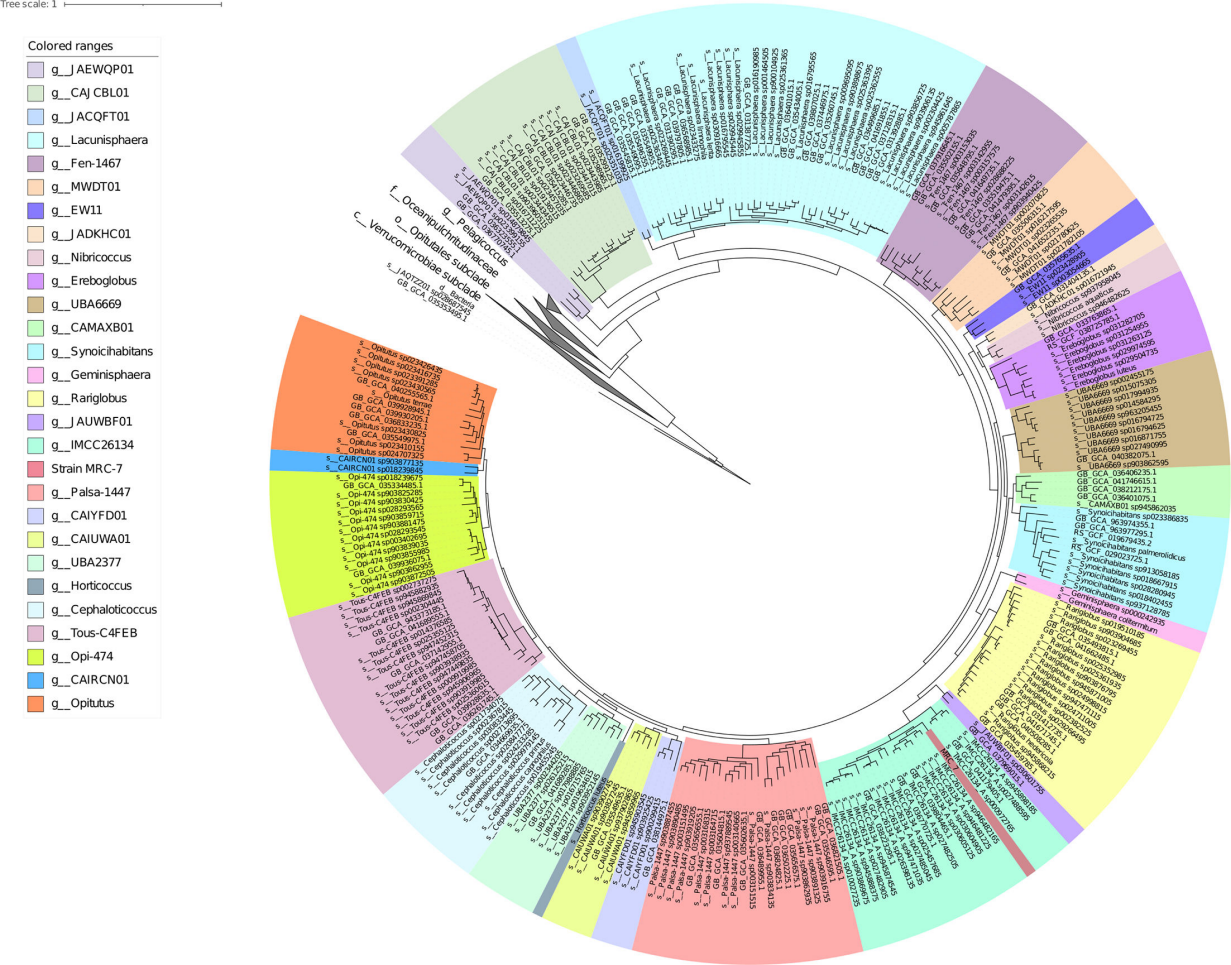
Phylogenetic placement of strain MRC-7 based on a concatenated alignment of bacterial 120 marker proteins, constructed using GTDB-Tk (v2.4.1) and pplacer (v1.1.alpha19-0-g807f6f3) (19) with GTDB reference data release r226. Taxonomic ranks are abbreviated as follows: p__, phylum; c__, class; o__, order; f__, family; g__, genus; s__, species. The scale bar represents the number of substitutions per site.

Since members of the family Opitutaceae are distributed in diverse environments but are difficult to cultivate (8, 9), genome analysis of its members would be valuable for elucidating their roles in ecosystems. Therefore, we hereby report the genome sequence of strain MRC-7. Genomic DNA of the strain was extracted from 7-day cultures in R2A broth (25°C) using DNAiso Reagent (TaKaRa). The genomic DNA was sheared to approximately 10–20 kbp using a g-TUBE device. The library was prepared with the SMRTbell Express Template Prep Kit 2.0 (PacBio) and sequenced on the Sequel IIe system (PacBio). Overhang adapter sequences were removed using SMRT Link (ver. 12.0.0.177059) to prepare subreads. HiFi reads (*n* = 17,157; *N*_50_, 17,658 bp) were obtained by aligning subreads and excluding those with an average quality score <20. The reads ≤1,000 bp were removed using Filtlong (ver. 0.2.1; https://github.com/rrwick/Filtlong), resulting in 15,572 reads used for assembly. Genome assembly was performed with Flye (ver. 2.9.1-b1780) (10), and circularity was confirmed using Bandage (ver. 0.8.1) (11). Phylogenetic position was confirmed with GTDB-Tk (12) using “classify_wf” command and visualized using iTOL (ver.7.2.1) (13). AAI was calculated using FastAAI (ver. 1) (14). Genome completeness and contamination were evaluated with CheckM (ver. 1.2.3), showing 100% completeness and 0% contamination (15). CDSs were annotated using DFAST (ver. 1.2.0) (16) and KEGG via GhostKOALA (ver. 3.1) (17). Carbohydrate-active enzymes were annotated using the dbCAN3 server (18). A phylogenetic tree based on the concatenated 120 bacterial marker proteins confirmed that MRC-7 belongs to the family Opitutaceae (Fig. 1). The strain is most closely related to *Rariglobus hedericola* (GCF_007559335), with 57.08% AAI, indicating that it represents a potentially novel genus within the family Opitutaceae. The genome consisted of a single chromosome of 4,342,534 base pairs with a G + C content of 65.2%. Annotation by DFAST revealed that the chromosome contained 3,428 CDSs, 49 tRNA genes, and 3 rRNA genes. The number of KEGG orthologs was 1,416 (41.3% of the total). Several CDSs were annotated as glycoside hydrolases (GHs) and polysaccharide lyases (PLs), both of which are involved in pectin degradation (Table 1).

**TABLE 1:** Detected enzyme classes involved with the assimilation of pectin

Activities	Enzyme classes
Degradation of:	
Homogalacturonan	GH2, GH16_3, GH30_5, GH43_4, and GH43_24
Type I rhamnogalacturonan	GH28, GH105, GH106, and GH138
Type II rhamnogalacturonan	GH2, GH105, GH127, GH140, and GH143
Cleavage of:	
Pectin	PL1 and PL9_1
Rhamnogalacturonan	PL11_1, PL9_1, and PL11
Unsaturated rhamnogalacturonan	PL11, PL11_1, and PL26

As strain MRC-7 was isolated from a duckweed co-cultivation system, the environment may have contained plant cell wall debris released during duckweed growth. Since plant cell walls contain various types of pectin (20), the detected enzymes may have enabled MRC-7 to assimilate pectin from duckweed.

## Data Availability

The complete genome sequence of the Opitutaceae bacterium strain MRC-7 was deposited via the DNA Data Bank of Japan under the accession number AP038926 (BioProject/BioSample PRJDB19889/SAMD00858014; DDBJ Sequence Read Archive DRA020536). The data annotated using the dbCAN3 server, the KEGG database, and FastAAI were deposited via Figshare (https://doi.org/10.6084/m9.figshare.29192756).
